# Antimicrobial Activity of Some Medicinal Herbs to the Treatment of Cutaneous and Mucocutaneous Infections: Preliminary Research

**DOI:** 10.3390/microorganisms11020272

**Published:** 2023-01-20

**Authors:** Alexandra Noites, Iara Borges, Bruno Araújo, Joaquim C. G. Esteves da Silva, Natália M. de Oliveira, Jorge Machado, Eugénia Pinto

**Affiliations:** 1 Laboratory of Applied Physiology, ICBAS—Institute of Biomedical Sciences Abel Salazar, University of Porto, 4099-002 Porto, Portugal; 2 Laboratory of Microbiology and Biological Sciences Department, FFUP—Faculty of Pharmacy of University of Porto, University of Porto, 4099-002 Porto, Portugal; 3 Research Center in Chemistry (CIQUP), Institute of Molecular Sciences (IMS), Department of Geosciences, Environment and Spatial Plannings, Rua do Campo Alegre s/n, 4169-007 Porto, Portugal; 4 CBSin—Center of BioSciences in Integrative Health, 4250-105 Porto, Portugal; 5 CIIMAR—Interdisciplinary Centre of Marine and Environmental Research, University of Porto, 4450-208 Porto, Portugal

**Keywords:** antimicrobial activity, *Azadirachta indica*, *Coptis chinensis*, *Leptospermum scoparium*, superficial infections

## Abstract

(1) Background: Superficial, including cutaneous and mucocutaneous infections are a current public health problem with universal distribution. One of the main concerns, in the present/future, is fungal/bacterial infections by resistant microorganisms. This study aimed to verify if decoctions of coptidis (*Coptis chinensis*, Ranunculaceae family), neem (*Azadirachta indica*, Meliaceae family), and their essential oils (EOs), as well as the EO of manuka (*Leptospermum scoparium*, Myrtaceae family) have antimicrobial activity against prevalent species of microorganisms responsible for superficial infections. (2) Methods: The antimicrobial activity was determined by the minimum inhibitory concentration (MIC), using broth microdilution method, and minimum lethal concentration (MLC) was determined from subculture of MIC plates. (3) Results: *C. chinensis* EO and decoction demonstrated some antifungal action against the yeasts and dermatophytes tested. Greatest bactericidal effect against *Propionibacterium acnes* and some action against *Staphylococcus aureus* was observed. For *A. indica* only EO proved activity against dermatophytes and *P. acnes*. *L. scoparium* EO showed the broadest antimicrobial spectrum with activity against bacteria, yeasts, and dermatophytes showing greater activity against *P. acnes* and *S. aureus*. (4) Conclusions: *C. chinensis* (EO/decoction), EOs of *L. scoparium* and *A. indica* proved in vitro efficacy against fungal, bacterial, or mixed agents of superficial infections, either by sensitive or resistant strains.

## 1. Introduction

Superficial infections, namely cutaneous and/or mucocutaneous understood in healthy individuals, they are often responsible for local symptoms in the skin or in the infected mucosae, are a current public health problem, and their epidemiology has recently shifted. Further, with the increase in immunodeficient individuals depending on its severity, may result in high morbidity and lower quality of life. A diverse phylogenetic array of microorganisms, whether it is yeast-like or filamentous fungi, Gram-positive or Gram-negative bacteria, are involved in these pathologies [1,2,3]. Among the most common superficial fungal infections are those caused by yeasts such as cutaneous or mucocutaneous candidiasis and pityriasis versicolor, and those caused by dermatophytes such as tinea which targets nails, skin, or hair. Within the potential pathogenic species in these groups are yeasts belonging to genus *Candida* with the prevalence of *Candida albicans* and *Malassezia* spp. [1,2,3]. Apropos of filamentous fungi dermatophytes, the genera involved are *Trichophyton*, *Microsporum*, *Arthoderma*, *Nannizia*, and *Epidermophyton* [3,4]. *Aspergillus* spp. is an ubiquitous and opportunistic filamentous fungi but generally does not cause disease in immunocompetent patients and are rarely involved in superficial infections [5,6]. Other fungal agents may be involved in this type of infection, alone or in combination with those described above, as well as the possibility of association with bacteria [7]. In bacterial cutaneous infections, the most common causative agent is Gram-positive, *Staphylococcus aureus*, frequently found in the human respiratory tract and on skin surfaces and often associated with dermatophytes in superficial infections [1,7]. The emergence of antibiotic resistant forms of pathogenic *S. aureus* (e.g., methicillin resistant *S. aureus* (MRSA)) is a worldwide issue in clinical medicine [1]. *Propionibacterium acnes*, another Gram-positive is associated with *acne vulgaris*, a skin disease characterized by the formation of comedones, papules, pustules, nodules, and/or cysts responsible for painful symptoms. It is one of the most prevailing dermatologic diseases in the world, affecting up to 80% of teenagers and frequently remains into adulthood [8,9]. Gram-negative bacteria as *Pseudomonas aeruginosa*, usually found in the environment, is a pathogen associated with infections in humans, being involved in septicemia and nosocomial pneumonia, especially in immunosuppressed individuals, and very resistant to antimicrobials [10]. It can spread via water and can be responsible for auditory, ocular, and skin rash. *Escherichia coli*, another Gram-negative bacteria fundamentally found in the environment, food, and gastrointestinal tract of humans and animals may not be so much associated with cutaneous or mucocutaneous pathology, though it serves as bacterial model widely used in any study of anti-bacterial activity [1]. One of the main concerns in the present, and in the future, lies in the emergence of resistance to the existing antimicrobials [1,2,11]. These resistances, often observed in bacteria, are also a reality in fungi, besides the fact that the number of antifungals available in the market is much lower compared with antibacterial drugs. The uncritical use of antibacterial drugs aggravates this picture, enabling the installation of fungal infections and co-infections by resistant bacteria [2,11]. For that, the search for new therapeutic modalities has increased requirements in natural medicinal therapy [1,12]. During the last years, essential oils (EOs), plant extracts, herbs, and other vegetable derivate products have been widely studied for their antimicrobial activity [13,14,15,16,17,18,19]. Skin conditions can be originated by one single pathogenic agent or more, for which it turns interesting to explore different plants (for possible studies of synergy) as well as diverse extraction methods without disregarding the rule of maintaining, when possible, an option with the solvent used in the traditional therapeutic preparation. In this case, water is the solvent present in traditional decoctions [13]. Several plants already traditionally used for their antimicrobial properties are being studied in such a way that their value is scientifically proven, and their anti-infectious potential can be ascertained alone or in synergy with classical antimicrobials [20,21,22,23,24]. A natural compound with high antimicrobial efficacy and fewer side effects is a desirable substitute or complement for chemical treatments having various adverse effects [2]. Plant-based and traditional medicines are used for the treatment of superficial infections, mainly in developing countries. Some plants are used by different traditional medicines: *Coptis chinensis* (coptidis), used in Traditional Chinese Medicine (TCM); *Azadirachta indica* (neem/nim, referred as neem) used by traditional Ayurveda medicine; and *Leptospermum scparium* (manuka) has been used historically by traditional Maori medicine. Main classic indications and more recent studies have demonstrated that *C. chinensis* has several pharmacological activities among which we highlight antibacterial, antifungal, antiviral, anti-atherosclerosis, antidiabetic, antiarrhythmic, anti-hypertension, anti-inflammatory, antioxidant, antitumor, anti-emetic, anti-diarrheal, toothache analgesic, prevention, and treatment of jaundice, among others [25]. Traditional Ayurveda medicine used *A. indica* in the treatment of several diseases; *A. indica* leaf and its constituents demonstrated immunomodulatory, anti-inflammatory, antihyperglycemic, anti-ulcer, antimalarial, anti-fungal, antibacterial, antiviral, antioxidant, antimutagenic, and anticarcinogenic properties [26,27]. *L. scoparium* has been used for a variety of infectious-related conditions including urinary tract, intestinal complaints, coughs, colds, and skin. *L. scoparium* EO is worldwide employed for its activity against Gram-positive bacteria, including antibiotic resistant strains and fungi [1,16,28]. In a previous study, our group found relevant antifungal effects of decoction and EO of *C. chinensis* and of *L. scoparium* EO against prevalent species of *Candida*, such as *C. krusei* and *C. glabrata*, as well as a particular potential of *L. scoparium* EO in the inhibition in germ tube formation which is considered a virulence property *C. albicans* [29]. So, the present work aims to further investigate the antimicrobial activity (minimum inhibitory concentration (MIC) and minimum lethal concentration (MLC)) to provide a scientific rationale for the ethnomedicinal use of the EO and decoctions of *C. chinensis*, *A. indica,* and *L. scoparium* EO on the treatment of superficial infections caused by a broader range of fungi (yeasts and filamentous fungi) and bacteria (Gram-positive and Gram-negative).

## 2. Materials and Methods

### 2.1. Essential Oils, Decoctions, Standards and Reagents

Decoctions samples of *C. chinensis* Franch. (coptidis rhizome decoction) and *A. indica* A. Juss. (nim/neem seeds decoction) were prepared from dried plants by adding 30 g of plant to 250 mL of distilled water, letting it soak for 45 min, and then simmering moderately for 30 min. After simmering, decoctions were allowed to stand to cool and were filtered. Stock solutions from samples of EOs and decoctions were prepared by dilution at 500.00 µL/mL (*v*/*v*) in dimethyl sulfoxide (DMSO, Sigma-Aldrich, St. Louis, MO, USA). Quality control was performed with the commercial voriconazole (kindly provided by Pfizer, New York, USA) for fungi and gentamicin (Sigma-Aldrich, St. Louis, MO, USA) for bacteria. Fluconazole (Sigma-Aldrich, St. Louis, MO, USA) and gentamicin were used as reference antifungal drugs. The stock solutions were also prepared in DMSO in order to obtain a concentration of 2 mg/mL. Dried plants and EO of *A. indica* seeds, *C. chinensis* rhizome and the EO of *L. scoparium* branches and twigs were acquired to Magnolien Apotheke, Germany, Karlsruher Str. 14, 69126; and kept at the Applied Physiology Laboratory (ICBAS-UP) with batch number BRR-3576/1-3. The EO was analyzed chemically by gas chromatography (GC) (Trace 1300 gas chromatography; Thermo Fisher Scientific, Waltham, MA, USA) coupled with a mass spectrometry (MS) system (ISQ single quadrupole mass spectrometer; Thermo Fisher Scientific) and the results are presented in Supplementary Tables S1–S3.

### 2.2. Microorganisms and Culture Media

The susceptibility tests were performed for yeasts such *C. albicans* (ATCC 10231) and *Malassezia furfur* (P26, clinical isolate); and the filamentous fungi: *Aspergillus fumigatus* (ATCC 204305) dermatophyte clinical isolates (*Trichophyton rubrum* FF5, *Microsporun canis* FF1 and *Epidermophyton floccosum* FF9). Regarding bacteria, susceptibility tests were performed against Gram-positive bacteria such as *S. aureus* (ATCC 25923 and a clinical MRSA strain) and *P. acnes* (ATCC 11827); and the Gram-negative *E. coli* (ATCC 25922) and *P. aeruginosa* (ATCC 27853). *Candida krusei* ATCC 6258 and *E. coli* ATCC 25922 reference strains were used for quality control.

All microorganisms were stored at −80 °C, in Mueller-Hinton broth ((MHB) Liofilchem, Roseto degli Abruzzi, Italy) with 15.0% glycerol for bacteria or Sabouraud dextrose broth ((SDB) bioMérieux, Marcy l’Etoile, France) with 20.0% glycerol for fungi. To ensure optimal growth conditions and purity, all strains were sub-cultured before each assay: *S. aureus*, *P. aeruginosa,* and *E. coli* on Mueller–Hinton agar (MHA, Liofilchem, Roseto degli Abruzzi, Italy) and incubated for 24 h, at 35 °C and *P. acnes* in MHA, anaerobiosis, at 35 °C, for three days; *Candida* species and *A. fumigatus* on Sabouraud dextrose agar ((SDA) bioMérieux, Marcy l’Etoile, France) and incubated at 35 °C for 24 h and 48 h, respectively; *M. furfur* on SDA with 2.0% of tween 80 (TW80), incubated at 35 °C for 3 days; dermatophytes on Mycosel agar (MYC, Becton Dickinson, Cockeysville, MD, USA) and incubated at 25 °C for 5–7 days.

The RPMI-1640 broth medium, with L-glutamine and without bicarbonate (Biochrom GmbH, Berlin, Germany), used on the evaluation of antifungal activity, was buffered with 0.165 mol/L of 3-(*N*-morpholino)-propanesulfonic acid ((MOPS) Sigma-Aldrich, St. Louis, MO, USA) and pH was adjusted to 7.0 ± 0.2 with 1.00 mol/L of NaOH (Panreac, Barcelona, Spain). For *M. furfur*, 2.0% of TW80 was added to RPMI medium. Mueller–Hinton broth 2 (MHB2, Becton Dickinson, Maryland, USA) was used on the evaluation of antibacterial activity. Particularly with MRSA, NaCl 2% was added to MHB2 medium.

### 2.3. Antimicrobial Susceptibility Testing by Broth Microdilution

The minimum inhibitory concentrations (MICs) and minimal lethal concentrations (MLCs) were used for determining the antimicrobial activity in agreement with the recommendations of the Clinical and Laboratory Standards Institute (CLSI) reference documents, with minor modifications: M27–A3 and M38–A2 for yeasts and filamentous fungi, respectively, and M100-S25 for bacteria [30,31,32,33].

Stock solutions of decoctions and EOs in DMSO were serial two-fold diluted within the concentrations range of 0.31–20.00 µL/mL (equivalent to 0.03–2.0%, *v*/*v*), in MHB2 for bacteria and RPMI for fungi. For the maximum final concentration of DMSO (2.0%), no interference on the bacterial/fungal growth was observed. Sterile microtiter plates of 96 flat bottom wells were used to evaluate the susceptibility of the microorganisms. Equal volumes of cell suspension and decoctions/EOs dilutions were added in each well.

A sterility control, a growth control, and a quality control (performed with *C. krusei* ATCC 6258 reference strain with voriconazole, 0.06–2.00 µg/mL, for antifungal activity and *E. coli* ATCC 25922 reference strain with gentamicin, 0.06–4.00 µg/mL, for antibacterial activity) were performed in each experiment. Results obtained for quality control were within the recommended limits. The tests with decoctions/Eos and reference drugs were prepared in duplicate and performed at least three times.

#### 2.3.1. Antibacterial Susceptibility Testing

Bacteria cell suspensions were prepared from pure cultures on MHA/24 h/3 days for *P. acnes*, in a sterile saline solution (API ampoules; BioMérieux, Marcy L’Etoile, France). The density was adjusted to obtain a MacFarland standard of 0.5 at 530 nm, corresponding to 1–5 × 10^6^ cells/mL (spectrophotometer, BioMérieux, Marcy L’Etoile, France). This suspension was diluted in MHB2 to obtain an inoculum suspension of 1–5 × 10^4^ CFU/mL. Equal volumes of cell suspension (50 µL) and decoctions/EOs dilutions (50 µL) were added in each well. The plates were incubated aerobically at 35 °C for 24 h and for 3 days in anaerobiosis for *P. acnes*. MIC was recorded as the lowest concentration at which no visible growth was observed. The MLC was assessed by spreading 20 µL of culture collected from wells showing no visible growth on MHA plates. The MLC was determined as the lowest concentration at which no colonies grew after 16–18 h incubation at 35 °C (3 days in anaerobiosis for *P. acnes*).

#### 2.3.2. Antifungal Susceptibility Testing

Yeast cells suspensions were prepared from pure cultures on SDA/24 h, or SAB plus TW80/3 days for *M. furfur*, in sterile saline solution and adjusted to MacFarland standard of 0.5 at 530 nm, corresponding to an initial suspension of 1–5 × 10^6^ cells/mL. The suspension was diluted at 1:50 and 1:20 in RPMI or RPMI + TW80, corresponding to a work suspension of 1–5 × 10^3^ CFU/mL. For filamentous fungi, a spore suspension is prepared from pure culture with spores in SDA (*A. fumigatus*) or MYC (dermatophytes), in sterile saline one drop of TW20 is added. The cell concentration was adjusted by the spore count in a Neubauer chamber and diluted in RPMI to obtain the acceptable inoculum (0.4–5 × 10^4^ CFU/mL for *A. fumigatus* and 1–3 × 10^3^ CFU/mL for dermatophytes). Equal volumes of cell suspension (100 µL) and decoctions/EOs dilutions (100 µL) were added in each well. The plates were incubated aerobically at 35 °C through 48 h for *C. albicans* and *A. fumigatus*, 3 days for *M. furfur* and at 25 °C for 5–7 days for dermatophytes. MICs were determined as the lowest concentrations resulting in 100.0% growth inhibition, compared with the control (decoctions/EO-free); an example is seen in supplementary Figure S1. The MLC was assessed by spreading 20 µL of culture collected from wells showing no visible growth on SDA, or SAB + TW80, plates. The MLC was determined as the lowest concentration at which no colonies grew after 48 h incubation at 35 °C for *C. albicans* and *A. fumigatus*, 3 days for *M. furfur,* and 7 days at 25 °C for dermatophytes: an example is seen in supplementary Figure S2.

### 2.4. Statistical Analysis

Susceptibility tests were carried out in duplicates within independent groups by at least three times and results are presented as mean values.

## 3. Results

Table 1 shows the results obtained for the antimicrobial activity of decoctions and EOs of *C. chinensis* and *A. indica*, and the EO of *L. scoparium* against the tested fungal and bacterial species.

*C. chinensis* EO and respective decoction, as well as *L. scoparium* EO showed the broad-spectrum antimicrobial activity with MIC values ranged from 0.63 to >20.00 µL/mL while *A. indica* EO exhibited a wider range of activity only against dermatophytes and *P. acnes* (Table 1).

### 3.1. Antifungal Effects

The results of MIC and MLC, obtained for the activity of the two decoctions and three EOs, against the six different fungal strains are present in Table 1.

*C. chinensis* EO and decoction showed a broad-spectrum antifungal activity: MIC values ranged from 5.00 to >20.00 µL/mL; higher activity was observed for *C. albicans* (MIC of 5.00 µL/mL); for the dermatophytes *E. floccosum* and *T. rubrum* as well as for *M. furfur* the MIC was of 20.00 µL/mL. The activity was similar between EO and decoction for all the species studied. The fungicidal effect showed that they are fungicidal for most of the susceptible strains, with an MLC equal or just one log_2_ dilution higher than MIC. *A. fumigatus* and *M. canis* were the most resistant filamentous fungi.

EO of *A. indica* showed activity only against dermatophytes while decoction of *A. indica* did not show antifungal activity in concentration equal to 20.00 μL/mL in the species tested.

Regarding *L. scoparium* EO, antifungal activity was demonstrated against almost all tested fungi, with the MIC and MLC values ranging from 5 to >20.00 µL/mL. The fungicidal effect with the MIC value were obtained for dermatophytes and *M. furfur*.

### 3.2. Antibacterial Effects

The results of MIC and MLC, obtained for two decoctions and three EOs, against the five different bacteria strains, are present in Table 1.

Considering *C. chinensis* EO and respective decoction, it was shown that they are bactericidal at the MIC, and both showed the greatest activity against *P. acnes* with the EO being more active (MIC of 0.63 µL/mL) than decoction (MIC of 1.25 µL/mL). Regarding *S. aureus* the MIC values were 20.00 µL/mL for both methicillin resistant and susceptible strains. Concerning *L. scoparium* EO, activity was also demonstrated against Gram-positive bacteria as *P. acnes* (MIC of 0.63 µL/mL) and *S. aureus* (MIC of 0.63–1.25 µL/mL), plus it can be underlined that MLC was equal to MIC for *P. acnes* (MLC of 0.63 µL/mL) and the effect only was bacteriostatic for *S. aureus* (MLC >20.00 µL/mL). Moreover, the MIC was lower for MRSA strain compared with ATCC strain of *S. aureus*. The EO of *A. indica* showed activity only against Gram-positive *P. acnes*, although with an MIC/MLC of 10.00/20.00 µL/mL, respectively. Nevertheless, no activity was observed for the *A. indica* decoction. Closing the discussion about the antimicrobial effects it is possible to highlight that the broadest antibacterial spectrum of activity was observed for *L. scoparium* EO and the more susceptible bacteria was *P. acnes*. Considering *E. coli* and *P. aeruginosa*, these Gram-negatives demonstrated a higher resistance to all the EOs, and decoctions studied, with MIC values >20.00 µL/mL.

## 4. Discussion

Cutaneous and mucocutaneous infections are a current public health problem with fungal skin infections as one of the most universally widespread forms. Bacterial superinfections might co-occur with fungal superficial infections, thus it is advantageous for antifungal agents to have antibacterial action as well [7]. New therapies to treat these infections ought to be seen as necessary due to their growing incidence and the development of resistance to conventional antimicrobials.

Plants’ parts, extracts, decoctions, infusions, and essential oils, largely used as cooking and flavoring agents, are more and more considered to have a wide spectrum of antimicrobial activity, mainly due to alkaloids, phenolic compounds, and terpenes, which have antibacterial and antifungal activity, as well as the ability to suppress a number of fungal virulence factors [34,35]. Herbal decoctions are specific combinations of different herbs blend as formulas based on the patient’s symptoms and characteristics, to treat several diseases including infections in traditional medicine since TCM has been scientifically proven to serve as an efficient complementary medicine to conventional drugs for a diversity of diseases [36,37]. EOs are natural products produced by aromatic plants, being terpenes and terpenoids they are mainly compounds with antimicrobial activity. Having a lipophilic nature, the EO can be integrated into membrane structures acting as a facilitator in cell permeability and as an enhancer in the inactivation of enzymes. Considering fungi, EO can act by ergosterol synthesis inhibition, which can also change cell membrane permeability, inhibit enzymes involved in cell wall synthesis which that can alter the morphology producing oxygen reactive species, and interact with the mitochondrial function [38,39]. Microbicidal activity is considered as a desirable quality for antimicrobial agents, since it can totally eliminate the microorganisms from the infected tissue, while bacterio-static or fungistatic activity poses the question of non-total elimination, particularly in immunocompromised patients. The fact that many EOs showed fungicidal activity at low concentrations, continues to encourage the search for more active compounds from plants [34,38,40,41].

*C. chinensis* is well-known for its anti-inflammatory, antioxidative, immunosuppressive, antiviral, antibacterial, and antifungal functions [25,42]. In this study, *C. chinensis* decoction and the respective EO exhibited activity against *C. albicans*. Regardless of the discrepancy of results found by several authors, it seems that *C. chinensis* can be evaluated as a potential alternative in treatment of candidiasis originated by some species of Candida. Even though the observed MIC of *C. chinensis* against *C. albicans* was 5.00 µL/mL, the EO has a relative advantage to fluconazole if we take into account its fungicidal effect, fluconazole is only fungistatic. Relatively to filamentous fungi, *A. fumigatus* demonstrated low susceptibility for both decoction and EO of *C. chinensis*, with an MIC > 20.00 µL/mL. Other authors reported a demonstration of antifungal activity for *A. niger* at MIC > 20.00 mg/mL for methanol extract and acetone extract, and >40 mg/mL for hot water extracts [43]. With respect to dermatophytes, other filamentous fungi very important in superficial mycoses, results exhibited a low susceptibility (MIC ≥ 20.00 µL/mL). By last, considering antibacterial activity of *C. chinensis*, the higher bactericidal capacity was observed against *P. acnes* (MIC = MLC = 0.63 µL/mL for EO and 1.25 µL/mL for decoction) with a selective effect. Gram-positive bacteria such as *S. aureus* demonstrated to be less susceptible with an MIC of 20.00 µL/mL, along with Gram-negative bacteria as *E. coli* and *P. aeruginosa* which registered a low susceptibility with MICs > 20.00 µL/mL. The effect was higher for Gram-positive, which can be justified by the differences observed in the cell wall/outer membrane structures [25]. Other authors described positive activity of *C. chinensis* against *Bacillus cereus* > *E. coli* > *P. aeruginosa* = *S. aureus* though these results should be compared with caution since the activity was determined by paper disc diffusion assay [43].

Facing the similarity of results between EO and decoction antimicrobial activities, it is logical to admit that the bioactive compounds correlated with the antimicrobial activity can be the same, adding the fact that to obtain both EO and decoctions, it is implied a process that requires temperature rising, steam distillation and simmering, respectively. The main active ingredients of *C. chinensis* are alkaloids, which are also their most abundant chemical components [25]. Berberine and palmatine chlorides are both isoquinoline quaternary alkaloids, isolated from *C. chinensis* as the major active compound and have been recognized by many pharmacological effects, including antimicrobial activity [25,36,44].

Agarwal et al. showed that *A. indica* EO was not effective against *C. albicans*, even at concentrations up to 3.0% (*v*/*v*), equivalent to 30.00 µL/mL [34]. It should be considered that different susceptibility was observed depending on the type of microorganisms. Thus, we verified that although the tested yeasts (*Candida* and *Malassezia*) did not show susceptibility to *A. indica* EO and decoction, the dermatophytes within the filamentous fungi, and Gram-positive bacteria *P. acnes* showed susceptibility with cidal effect for concentrations of 0.5 to 2.0%. Making a comparison between the activity of *A. indica* decoction and EO, we can conclude that what justifies the activity of the EO is not present in the decoction, or if so, it is not in sufficient quantity, no activity was observed for the decoction at concentration up to 2.0% considering all the tested strains. In fact, the chemistry composition is variable between the different parts of the plant, the different kind of extracts, or different preparation methods and for that, it might produce different biological activity or strength [37]. Antifungal and antibacterial activity has been described for seed, bark, and leaves of *A. indica* extracts and for some of the compounds isolated from fruit coats and for *A. indica* oil from fresh twigs [27,45]. Although, aqueous extracts from *A. indica* leaves showed the capacity to suppress mycelial growth, the activity for ethanolic and ethyl acetate extracts was higher, being the strongest inhibition of fungal growth obtained with ethyl acetate at 20.0% [45].

*L. scoparium* EO showed a spectrum and an antifungal activity that is not far from *C. chinensis* (decoction or EO); on the other hand, the activity was higher against bacteria, compared with fungi, unlike *C. chinensis*. Considering the yeast genera tested with this EO, *M. furfur* showed more susceptibility (5.00 µL/mL) than *C. albicans* and it also was fungicidal for the experimented strain of *M. furfur*. Regarding our results, higher antimicrobial activity was scored against bacteria in comparison with yeasts, confirming the results in the studies of other authors [28,46,47]. Chen et al. obtained higher MICs for *C. albicans* (3.1%, *v*/*v* solution), *M. furfur* (1.6%), and *S. aureus* (10.0%) and Van Vuuren et al. showed for *S. aureus* an MIC of 4.00 mg/mL and for *P. acnes* of 1.00 mg/mL, higher than the values obtained in our study, of 1.25 and 0.63 µL/mL, respectively [1,28]. On the other hand, the same authors observed an MIC value for *C. albicans* of 8.00 mg/mL, lower than that observed in the current study for the ATCC strain (20.00 µL/mL) [28]. The results of the present work showed the highest activity for Gram-positive bacteria, dermatophytes, and *M. furfur*, which is in line with Christoph et al., which considered that the triketone content in the *L. scoparium* oil is responsible for the best inhibitory effects on Gram-positive bacteria and dermatophytes [28,48]. Furthermore, it is possible to confirm in our study that at least two ketones were found in considerable relative amount-5-cyano-2,4-dioxo-3-aza-spiro [5.5]undecane-1-carbothioic acid (14.51%) and 1,1′-(5-hydroxy-2,2-dimethyllbicyclo [4.1.0]heptane-1,7-diyl) bis-ethanone (5.75%), which might add to support the line of thought of Christoph et al. (Table S3).

Gram-negative bacteria showed resistance to *L. scoparium* EO (MIC > 20.00 µL/mL) in comparison with Gram-positives (MIC 0.63–1.25), with other authors confirming this data. In a review by Mathew C. et al., 2020, the authors refer that Song et al. showed MIC > 8.0% (*v*/*v*) for *P. aeruginosa* and >2.0% for *E. coli*, considering that the outer membrane of Gram-negative bacteria reduces the cell permeability, and the addition of Tris-EDTA increases the activity of the EO against these bacteria [49]. To justify the differences between different authors, we can consider the use of different microorganisms strains, the technical differences in the protocol of anti-microbial activity evaluation, and the variable chemical composition of EO or extracts. The composition of tested plant products, and consequently the antimicrobial activity, varies in function of the botanical source, geographical origin of plants with widely differing climatic and altitudinal zones, environmental conditions, parts of the plant used, season of harvest, several chemotypes, and the extraction method used [39,46].

## 5. Conclusions

TCMs can be used as complementary therapy with the standard antifungal drugs against fungal pathogens once that synergy or additive effects have been observed for plant combinations as well as for combinations between plants extracts and conventional antifungal drugs [28,37,43]. *C. chinensis* (EO/decoction), *L. scoparium,* and *A. indica* EOs have shown preliminary potential regarding in vitro efficacy for future research and so verify a viable co/treatment of fungal, bacterial, or mixed superficial infections, either by sensitive or resistant strains. This study was the second step on the quest of the fascinating and promising world of ancient herbs within traditional use and in the future, it would be interesting to see the biochemical composition of the respective decoctions, analyze antifungal activity of decoctions and EO against the mycelia growth, and broaden the type of assays to assess antimicrobial activity and possible synergy of different extracts. The knowledge of traditional herbal medicine is fundamental for enriching the (re)discovery of new therapeutic agents. Studies in this area of research are very important to understand the role of the traditional application of many plants and to build a solid partnership between allopathic and traditional medicines.

## Figures and Tables

**Table 1:** Antimicrobial activity (minimum inhibitory and lethal concentration, MIC/MLC) of *Coptis chinensis* and *Azadirachta indica* decoctions/EO and *Leptospermum scoparium* EO.

		*C. chinensis* Decoction		*C. chinensis* EO		*A. indica* Decoction		*A. indica* EO		*L. scoparium* EO		Fluconazole		Gentamicin	
		MIC ^a^	MLC ^b^	MIC ^a^	MLC ^b^	MIC ^a^	MLC ^b^	MIC ^a^	MLC ^b^	MIC ^a^	MLC ^b^	MIC ^c^	MLC ^d^		
Fungi	*Candida albicans* ATCC 10231 *	5.00	10.00	5.00	10.00	>20.00	>20.00	>20.00	>20.00	20.00	>20.00	2.00	>128.00	ND	ND
	***Malassezia furfur* P26**	20.00	>20.00	20.00	20.00	>20.00	>20.00	>20.00	>20.00	5.00	5.00	ND	ND	ND	ND
	***Aspergillus fumigatus* ATCC 204305**	>20.00	>20.00	>20.00	>20.00	>20.00	>20.00	>20.00	>20.00	>20.00	>20.00	≥128.00	>128.00	ND	ND
	***Trichophyton rubrum* FF5**	20.00	≥20.00	≥20.00	≥20.00	>20.00	>20.00	5.00	5.00	5.00	5.00	16.00	64.00	ND	ND
	***Microsporum canis* FF1**	>20.00	>20.00	>20.00	>20.00	>20.00	>20.00	10.00	≥20.00	10.00	10.00	8.00	32.00	ND	ND
	***Epidermophyton floccosum* FF9**	20.00	≥20.00	20.00	≥20.00	>20.00	>20.00	5.00	10.00	5.00	5.00	16.00	16.00	ND	ND
Bacteria	***Propionibacterium acnes* ATCC 11827**	1.25	1.25	0.63	0.63	>20.00	>20.00	10.00	20.00	0.63	0.63	ND	ND	ND	ND
	***Staphylococcus aureus* ATCC 25923**	20.00	>20.00	20.00	>20.00	>20.00	>20.00	>20.00	>20.00	1.25	>20.00	ND	ND	0.50	ND
	***Staphylococcus aureus* MRSA**	20.00	>20.00	20.00	>20.00	>20.00	>20.00	>20.00	>20.00	0.63	>20.00	ND	ND	ND	ND
	***Escherichia coli*** **ATCC 25922**	>20.00	>20.00	>20.00	>20.00	>20.00	>20.00	>20.00	>20.00	>20.00	>20.00	ND	ND	1.00	ND
	***Pseudomonas aeruginosa*** **ATCC 27853**	>20.00	>20.00	>20.00	>20.00	>20.00	>20.00	>20.00	>20.00	>20.00	>20.00	ND	ND	ND	ND

EO: essential oil; ^a^ MIC was determined by microdilution method and expressed in µL/mL (*v*/*v*); ^b^ MLC was expressed in µL/mL (*v*/*v*). Fluconazole was used as a reference antifungal drug and gentamicin as a reference antibacterial drug; ^c^ MIC was expressed in µg/mL (*w*/*v*). ^d^ MLC was expressed in µg/mL (*w*/*v*); ND: not determined. * Data published [29].

## Data Availability

Not applicable.

## Supplementary Materials

The following supporting information can be downloaded at: https://www.mdpi.com/article/10.3390/microorganisms11020272/s1, Table S1: List of the main detected compounds of the *Coptis chinensis* EO by GC/MS. Relative composition is shown by waning order. Table S2: List of the main detected compounds of the *Azadirachta indica* EO by GC/MS. Relative composition is shown by waning order. Table S3: List of the main detected compounds of the *Leptospermum scoparium* EO by GC/MS. Relative composition is shown by waning order. Figure S1: Minimum inhibitory concentration (MIC) determined by microdilution method (decoction v/v)/yeast. Figure S2: Minimum lethal concentration (MLC) from MIC plate (decoction v/v)/yeast.
