# Supplementary material for: Antimicrobial Activity of Some Medicinal Herbs to the Treatment of Cutaneous and Mucocutaneous Infections: Preliminary Research
Source: Microorganisms. 2023 Jan 20;11(2):272. doi: 10.3390/microorganisms11020272 (PMC9962950; doi:10.3390/microorganisms11020272)

Table S1: List of the main detected compounds of the Coptis Chinensis EO by GC/MS. Relative composition is shown by waning order.

Compound	Nature	Retention time	Relative %
Glycerol (3TMS derivate)	Fatty acid	13.69	62.33
---	Terpene	11.68	12.52
Butane-1,2,3-triol	Alcohol	12.47	11.39
3-hidroxy-3-metilbutanoic acid	Carboxylic acid	11.1	6.77
1,3-butanodiol	Alcohol	12.19	5.49
Glyceric acid (3TMS derivate)	Glyceric acid	16.37	1.45
1,4-dibutil (2R,3R)-2,3-dihidroxybutanodioate	Carboxylic acid	16.24	1.10

Table S2: List of the main detected compounds of the Azadirachta indica EO by GC/MS. Relative composition is shown by waning order.

Compound	Nature	Retention time	Relative %
**Esther of trans-9-octadecenoic acid**	Fatty acid	31.97	48.96
**Esther of hexadecanoic acid**	Fatty acid (Palmitic acid)	28.83	7.83
**9,12,15-Octadecatrienoic acid ethyl ester**	Fatty acid	40.19	7.59
**Esther of α-sitosterol**	Steroid	59.96	6.35
**1,1'-Dimethoxy--1,1',2,2'-tetrahydro-ψ,ψ-carotene**	Terpene	63.72	5.79
**Esther of octadecanoic acid**	Fatty acid (Stearic acid)	32.38	4.23
**---**	Terpene	54.70	2.41
**Esther of (Z,Z)-9,12-octadecadienoic acid**	Fatty acid (Linoleic acid)	31.88	2.34
**BSTFA (GC-MS derived)**	---	7.52	2.33
**Ethyl Iso-alcolate**	Steroid	55.85	2.30
**Squalene**	Terpene	42.22	2.22
**Stigmasterol**	Steroid	57.16	1.93
**BSTFA (GC-MS derivation)**	---	8.41	1.57
**Glycerol (3TMS derived)**	Fatty acid	13.66	1.33
**propylhester of hexadecanoic acid**	Fatty acid	38.02	1.03

Table S3: List of the main detected compounds of the Leptospermum scoparium EO by GC/MS. Relative composition is shown by waning order.

Compound	Nature	Retention time	Relative %
**trans-calamene**	sesquiterpene	27.68	15.38
**5-cyano-2,4-dioxo-3-aza-spiro[5.5]undecane-1-carbothioic acid**	ketone	29.72	14.51
**8,9-epoxy-neoisolongifolene**	sesquiterpene	27.13	7.86
**1,1'-(5-hydroxy-2,2-dimethyllbicyclo[4.1.0]heptane-1,7-diyl) bis-ethanone**	ketone	27.93	5.75
**1,2,3,5,6,8-α-hexahydro-4,7-dimethyl-1-(1-methylethyl)-(1S-cis)-naphthalene**	sesquiterpene	26.13	5.57
**α-copaene**	sesquiterpene	27.84	5.49
**[4aR-(4aà,7à,8aa)]-decahydro-4a-methyl-1-methylene-7-(1-methylethenol)-naphthalene**	sesquiterpene	26.97	5.42
**α-copaene**	sesquiterpene	24.43	5.38
**(1S-cis)-1,2,3,5,6,8a-hexahydro-4,7-dimethyl-1-(1-methylethyl)-naphthalene**	sesquiterpene	26.57	4.97
**α-Copaene**	sesquiterpene	23.74	4.45

**Caryophyllene**	sesquiterpene	25.48	2.85
**decahydro-1,1,7-trimethyl-4-methylene-1H-cycloprop[e]azulene**	sesquiterpene	25.91	2.20
**cubedol**	sesquiterpene	30.20	2.08
**4-epi-cubedol**	sesquiterpene	29.89	1.83
**α -pinene**	sesquiterpene	12.84	1.33
**[1aR-(1aà,4à,4aá,7bà)]-1a,2,3,4,4a,5,6,7b-octahydro-1,1,4,7-tetramethyl-1H-cycloprop[e]azulene**	sesquiterpene	25.21	1.18
**[1S-(1à,2á,4á)]-1-ethenyl-1-methyl-2,4-bis(1-methylethenyl)-cyclohexane**	sesquiterpene	24.72	1.09
**α-calacorene**	sesquiterpene	28.10	1.07

Figure S1: Minimum inhibitory concentration (MIC) determined by microdilution method (decoction v/v)/yeast.

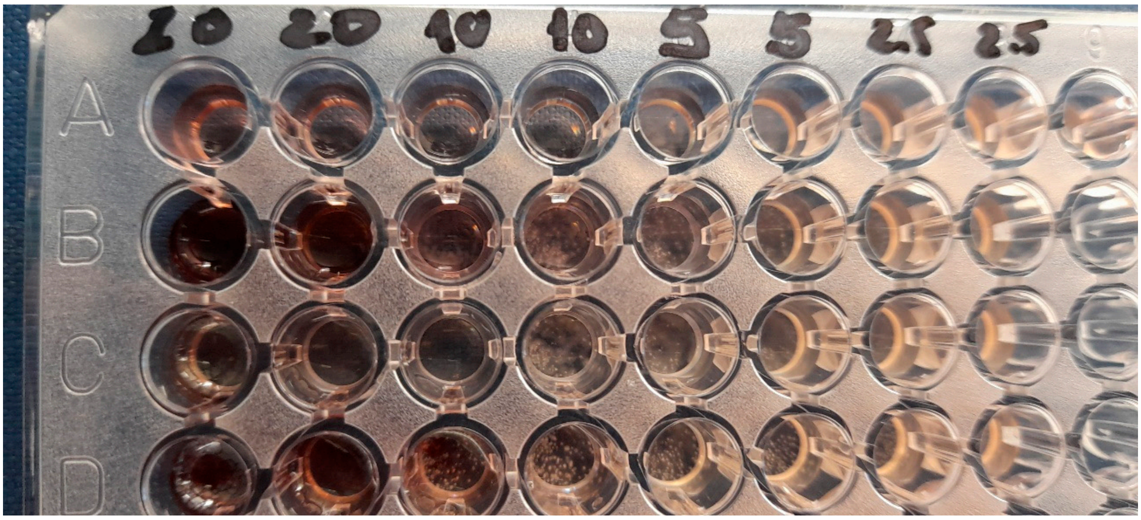

Figure S2: Minimum lethal concentration (MLC) from MIC plate (decoction v/v)/yeast.

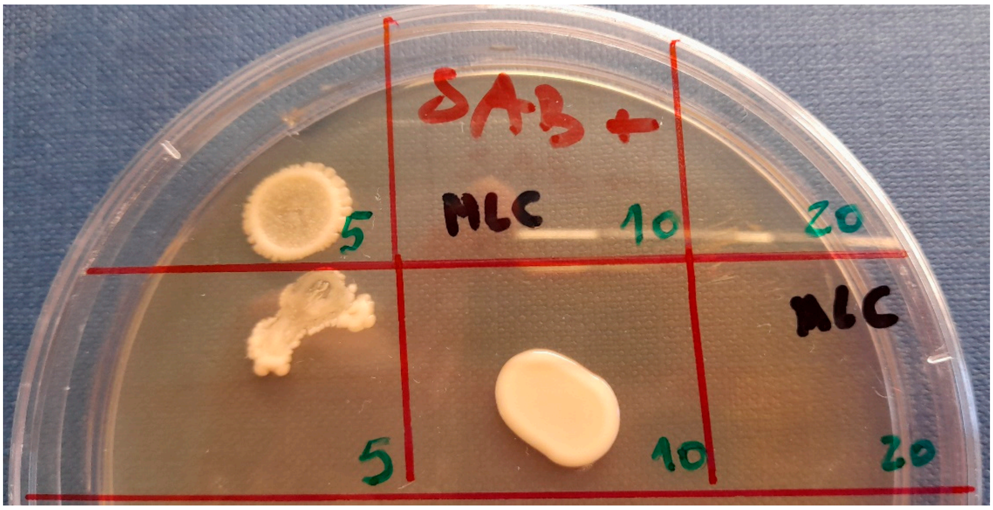

Supplement: Supplementary file 1 [file microorganisms-11-00272-s001.zip › microorganisms-2116519-supplementary.pdf]
